# Healthy lifestyle discussions between healthcare providers and older cancer survivors: Data from 12 cancer centers in the Southeastern United States

**DOI:** 10.1002/cam4.2568

**Published:** 2019-09-30

**Authors:** Karina I. Halilova, Maria Pisu, Andres Azuero, Courtney P. Williams, Kelly M. Kenzik, Grant R. Williams, Gabrielle B. Rocque, Michelle Y. Martin, Elizabeth A. Kvale, Wendy Demark‐Wahnefried

**Affiliations:** ^1^ UAB Division of Preventive Medicine University of Alabama at Birmingham (UAB) Birmingham AL USA; ^2^ UAB Comprehensive Cancer Center Birmingham AL USA; ^3^ UAB Department of Nutrition Sciences University of Alabama at Birmingham (UAB) Birmingham AL USA; ^4^ UAB School of Nursing University of Alabama at Birmingham (UAB) Birmingham AL USA; ^5^ UAB Division of Hematology & Oncology University of Alabama at Birmingham (UAB) Birmingham AL USA; ^6^ University of Tennessee Health Science Center Memphis TN USA; ^7^ University of Texas at Austin Austin TX USA

**Keywords:** cancer, cancer survivorship, health promotion, healthy lifestyle counseling, older adults

## Abstract

**Background:**

Little is known about the prevalence of healthy lifestyle (HLS) discussions between providers and older cancer survivors.

**Methods:**

We utilized cross‐sectional data from older cancer survivors (≥65 years) seen at 12 southeastern cancer centers during 2013‐2015. Data on demographics, time since diagnosis, weight, height, and healthy behaviors were collected. Respondents were asked if providers (oncologists, other physicians, and/or nurses) discussed exercise, healthy diet, weight management, and/or smoking cessation during clinical encounters. Descriptive statistics and bivariate associations between HLS topics and survivor characteristics were calculated.

**Results:**

Among 1460 cancer survivors, mean age was 74 years (SD 6), most were white (81%), and >1 year postdiagnosis (84%). The majority (71%) reported discussing at least one of three HLS topics (exercise 49%, healthy diet 53%, vegetable consumption 28%); 17% received counseling on all three. Weight loss was recommended to 33% of overweight/obese survivors and smoking cessation to 85% of current smokers. Oncologists and nurses discussed HLS less frequently compared to other physicians. Younger survivors (65‐74 years) received recommendations for exercise, weight loss, and tobacco cessation more often than older survivors (≥75 years). Compared to white respondents, minorities reported discussions on all topics more often except for tobacco cessation. Excluding tobacco cessation, survivors with recent cancer diagnoses (<1 year) reported HLS discussions more often than survivors >1 year postdiagnosis.

**Conclusion:**

Despite the American Cancer Society's recommendations, older survivors reported a low prevalence of HLS discussions with their providers, with some variation by demographic groups. Strategies are needed to promote these important discussions in this population.

## Graphical abstract

Provider's recommendations for weight management, healthy diet, physical activity, and smoking cessation are key in promoting healthy lifestyles (HLS) among older cancer survivors. However, we found that not all survivors discuss all relevant HLS with their providers, with some groups of survivors having these discussions less often than others. Additional studies and strategies are needed to promote HLS discussions and achieve the American Cancer Society's (ACS) recommended HLS goals especially for diet, weight, and physical activity among older cancer survivors in the Southeastern US healthcare system

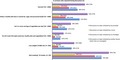

## 1 INTRODUCTION

Long‐term benefits of healthy behaviors among individuals diagnosed with cancer and other chronic diseases are well‐known.^1^ Healthy lifestyle (HLS) discussions initiated by physicians, especially oncologists, are powerful catalysts of change^2^, ^3^, ^4^ and could help promote healthy behaviors among cancer survivors. Since 1991, the American Cancer Society (ACS) has issued diet and exercise guidelines and encouraged oncology care teams to counsel survivors on HLS.^5^, ^6^, ^7^ The American Society of Clinical Oncology's (ASCO) endorsement of the need for oncology care teams to address weight control, diet, and physical activity with their patients reinforced this need.^8^, ^9^ However, these counseling encounters are not as frequent as they should be,^10^, ^11^, ^12^, ^13^, ^14^ and to what extent they occur in the clinical oncology setting is unclear. Only a few single institution studies have reported about HLS conversations in the oncology setting.^13^, ^14^ Moreover, literature suggests that there may be differences in which survivor groups are likely to receive HLS counseling.^11^, ^13^

It is particularly important for older cancer survivors to receive appropriate survivorship care, which includes HLS counseling.^15^ This growing population of cancer survivors^16^ experiences dual vulnerabilities due to both the impact of cancer itself and associated age‐related changes (eg decreasing physical activity, increasing frailty).^17^ Furthermore, older cancer survivors are high utilizers of the healthcare system.^17^, ^18^ Thus, it is important to effectively incorporate physician HLS recommendations during clinic visits to promote behaviors that minimize the impact of cancer (eg cancer recurrence, secondary obesity‐related cancers, mortality), decrease the occurrence of comorbidities,^8^, ^19^, ^20^ and improve overall health‐related quality of life^21^ in this population. Currently, limited data exist on HLS counseling frequency among older individuals with cancer,^10^, ^11^, ^12^, ^13^, ^14^ especially when such counseling is provided by oncologists and nurses.

To address these knowledge gaps, we analyzed data from a large, multicenter survey to describe the occurrence of HLS discussions among older cancer survivors. We report on HLS discussion type, frequency, and discussant (oncologists, another physician, and/or nurses). Moreover, we identify which patient groups more or less often have these discussions. Further evaluation of the missed opportunities for HLS counseling delivery across provider types and patient groups may assist clinicians, researchers, policy makers, and public health administrators in designing effective and better targeted supportive and survivorship care services for older adults with cancer.

## 2 METHODS

### 2.1 Study design and population

This cross‐sectional study utilized survey data from November 2013 to June 2015 among older cancer patients and survivors receiving oncology care within the University of Alabama at Birmingham (UAB) Health System Cancer Community Network (CCN). The UAB CCN is comprised of 12 academic and community cancer centers in Alabama, Georgia, Tennessee, Mississippi, and Florida.^22^ Cancer survivors were identified via hospital registries. Participants were older adults (≥65 years) with cancer diagnosed after 1 January 2008. The Institutional Review Board (IRB) of UAB and other sites approved this study. All study participants provided verbal informed consent prior to participation in telephone surveys.

### 2.2 Telephone surveys

Trained interviewers contacted 3106 patients by telephone, and 1460 (47%) respondents completed surveys. Main survey items analyzed in this study included HLS discussions with the healthcare provider, weight, height (BMI was calculated from self‐reported weight and height), smoking status, demographics (age, gender, race, marital status), socioeconomic status (SES; education, retirement status, sufficient income to cover basic needs), symptoms (measured with the MD Anderson Symptom Inventory [MDASI])^23^ and healthy behaviors (Godin Leisure‐Time Physical Activity [LTPA; ≤150 weekly minutes vs ≥150 weekly minutes]).^24^ These instruments have documented use in a broad range of patient populations, including older adults and individuals with cancer. Respondents also reported if they were on active treatment at the time of the survey. Cancer characteristics (type and stage) were abstracted from hospital tumor registries. Comorbidity score (NCI Comorbidity Index) was abstracted from Medicare claims data from 2012 to 2015.^25^

### 2.3 Outcomes

Our main outcome for this study was the report of HLS discussions with healthcare providers. The survey questions were developed in line with ACS guidelines on HLS counseling (Table S1), and were included in a larger survey questionnaire available from the corresponding author upon request. Respondents were asked if a member of their healthcare team provided HLS counseling at any time during and postcancer care on the following topics: (a) exercise, (b) following a healthy diet (low in animal fat, sugar, processed foods), (c) eating ≥ 5 servings of vegetables a day, (d) weight loss (if overweight or obese; BMI ≥ 25), or (e) smoking cessation (if currently smoking). Respondents also reported on the type of healthcare team member providing HLS counseling: response options were oncologist, another doctor, and/or a nurse. For the topics of exercise, healthy diet, and vegetables consumption, we combined responses to obtain the following outcomes: (a) having discussed at least one of the three topics (1‐of‐3), and (b) having discussed all three (all‐3).

### 2.4 Statistical analysis

Descriptive statistics (proportions, means, ranges, measures of central tendency) described overall sample characteristics. We first calculated the frequency of respondents reporting HLS discussion on each topic, at least 1‐of‐3, and all‐3, and by provider type. We used bivariate analyses to determine differences in the proportion of respondents having HLS discussions by demographics, SES, disease characteristics, comorbidity scores, symptoms, BMI, LTPA and smoking. SAS software (SAS v9.4, SAS Institute, Cary, NC) was used to conduct all analyses. Results were considered statistically significant at *P *≤ .0275 equivalent to a 10% False Discovery Rate level.^26^

## 3 RESULTS

### 3.1 Survivor demographics

Among the 1460 surveyed participants, mean age was 74 years, (SD 6), and most were female (60%), white (81%), and college‐educated (62%; Table 1). The most prevalent cancer types were breast (24%) and prostate (13%); a majority (84%) were >1 year postdiagnosis, and 28% were receiving active cancer treatment. With an average BMI of 28 (SD 6), most respondents were overweight (36%) or obese (28%). Less than 20% exercised at least 150 minutes per week and 6% were current smokers.

**Table 1:** Characteristics of cancer survivors surveyed on healthy lifestyles (HLS) discussions at 12 cancer centers in Southeastern US from November 2013 to June 2015 (N = 1460)

All	Total sample (N = 1460)	Discussed at least 1‐of‐3 HLS topics (exercise, healthy diet, or vegetables) 71%, (N = 1036)	*P*‐value
	Column % (n)	Row % (n)	
Age (y)			
Mean (SD)	74 (5.7)	74 (5.6)	—
Range	65‐99	65‐94	
Age group			
65‐74 y	58% (843)	73% (620)	.011
≥75 y	42% (617)	67% (416)	
Gender			
Female	60% (875)	71% (417)	.824
Male	40% (585)	71% (619)	
Race/ethnicity			
White	81% (1189)	69% (820)	<.001
Minority	19% (271)	80% (216)	
Education			
≤High school education	38% (553)	71% (393)	.943
Some college or higher	62% (907)	71% (643)	
Employment status			
Retired	84% (1223)	71% (863)	.450
Marital status			
Married	63% (918)	71% (649)	.766
Other	37% (539)	71% (385)	
Income status			
Sufficient income to meet basic needs	89% (1302)	71% (919)	.365
Cancer diagnosis			
<1 y	16% (232)	80% (186)	<.001
≥1 y	84% (1228)	69% (850)	
Cancer type			
Breast	24% (347)	73% (252)	.790
Prostate	13% (189)	69% (131)	
Lung	12% (169)	68% (115)	
Hematologic	11% (165)	72% (120)	
Gynecologic	9% (140)	69% (97)	
Colorectal	7% (109)	73% (80)	
Head and Neck	4% (66)	77%(51)	
Other	19% (275)	69% (190)	
Cancer stage at diagnosis			
Stage 0‐II	57% (834)	72% (599)	.1004
Stage III‐IV	25% (371)	73% (270)	
Missing	18% (255)	65% (167)	
Treatment status			
On active treatment	28% (411)	73% (298)	.4150
Comorbidity Score			
0‐1	63% (915)	69% (627)	.023
≥2	35% (514)	75% (384)	
Missing	2% (31)	81% (25)	
Symptoms			
Fatigue	83% (1215)	72% (875)	.047
Pain	59% (866)	72% (627)	.143
Breathing problems	55% (799)	72% (578)	.201
Distress	51% (740)	73% (541)	.067
Most common symptom management source used			
Traditional healthcare (physicians, nurses)	50% (724)	72% (521)	.432
Nontraditional care (healers, naturopaths, herbalists, friends/family, etc)	17% (242)	69% (166)	
Other	9% (136)	75% (102)	
No help	25% (359)	69% (247)	
Body Mass Index—Mean (SD)	28 (5.8)	29 (5.9)	
Underweight (<18.5)	3% (36)	64% (23)	.009
Normal weight (18.5‐24.9)	34% (490)	68% (332)	
Overweight (25.0‐29.9)	36% (529)	70% (368)	
Obese (≥30)	28% (405)	77% (313)	
Godin Leisure‐Time Physical Activity (LTPA), Moderate or Vigorous Physical Activity			
<150 min per week	81% (1184)	70% (829)	.100
≥150 min per week	19% (276)	75% (207)	
Current smoker	6% (92)	64% (59)	.134

Note

*P* values ≤ .028 are significant at a 10% False Discovery Rate level.

### 3.2 HLS topics discussed

**Figure 1:**
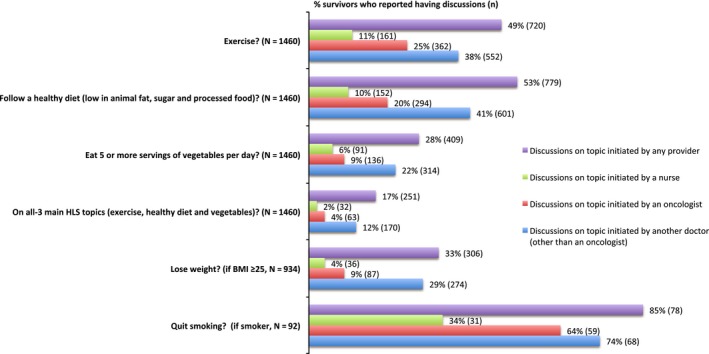
Frequency of cancer survivors reporting healthy lifestyle (HLS) discussions by discussion and provider type (N = 1460). *“*At any time during your cancer care and after, has your provider, or anybody else on your care team, advised you to…”

Overall, 71% reported discussing at least 1‐of‐3 HLS topics: exercise, healthy diet, or vegetable consumption (Table 1). One half reported being advised to exercise (49%) or follow a healthy diet (53%), whereas approximately one‐quarter (28%) were encouraged to eat more vegetables. Only 17% reported discussing all‐3 HLS topics (Figure 1). Among overweight or obese survivors, one‐third was advised to lose weight, and almost all smokers were advised to quit smoking (Figure 1).

### 3.3 HLS discussions by provider type

Most participants reported having HLS discussions with a doctor other than their oncologist or nurse (Figure 1). Oncologists provided HLS counseling more often than nurses, with exercise counseling proportions of 25% vs 11%; diet counseling proportions of 20% (9% for vegetables) vs 10% (6% for vegetables); and all‐3 counseling proportions of 4% vs 2%, respectively. Among overweight or obese participants, 9% reported discussing weight loss with oncologists and 4% with nurses.

### 3.4 Survivor characteristics by receipt of HLS discussions

Younger survivors reported having HLS discussions regarding exercise, weight loss, and smoking cessation more often as compared to older survivors (Table 2). Non‐white respondents reported having HLS discussions regarding exercise, healthy diet, vegetable consumption, and weight loss more often than white respondents. Survivors more proximal to their cancer diagnosis reported HLS discussions more often on all topics except for smoking cessation. No statistically significant and/or meaningful differences were observed by gender, SES, cancer type, cancer stage, comorbidity score, symptoms, or LTPA.

**Table 2:** Bivariate analysis between cancer survivor characteristics and receipt of healthy lifestyles (HLS) discussions (N = 1460)

	Exercise N = 1460	Follow a healthy diet (low in animal fat, sugar and processed food) (N = 1460)	Eat five or more servings of vegetables per day (N = 1460)	Discussed all‐3 main HLS topics (exercise, healthy diet, and vegetables) (N = 1460)	Lose weight, (if BMI ≥ 25) 64% (N = 934)	Quit smoking (If smokers) 6% (N = 92)
	Row % (n) *P*					
Age group						
65‐74 y	54% (457) <.001	56% (468) .053	30% (253) .047	19% (164) .007	36% (207) .003	90% (62) .019
≥75 y	43% (263)	50% (311)	25% (156)	14% (87)	27% (99)	70% (16)
Gender						
Female	49% (428) .708	54% (474) .445	30% (262) .045	19% (166) .028	32% (167) .482	84% (38) .930
Male	50% (292)	52% (305)	25% (147)	15% (85)	34% (139)	85% (40)
Race/ethnicity						
White	47% (560)	52% (615)	26% (307)	16% (186)	31% (226)	86% (63) .427
Minority	59% (160) <.001	61% (164) .009	38% (102) <.001	24% (65) .001	41% (80) .008	79% (15)
Education						
≤High school education	47% (259) .139	52% (285) .277	30% (167) .147	17% (156) .992	34% (116) .668	80% (41) .191
Some college or higher	51% (461)	54% (494)	27% (242)	17% (95)	32%(190)	90% (37)
Cancer diagnosis						
<1 y	60% (140) <.001	59% (138) .041	34% (80) .017	22% (52) .021	41% (62) .015	58% (7) .006
≥1 y	47% (580)	52% (641)	27% (329)	16% (199)	31% (244)	89% (71)
Cancer type						
Breast	51% (178) .590	56% (193) .469	30% (105) .416	19% (65) .595	36% (84) .429	86% (12) .421
Prostate	52% (99)	51% (96)	23% (44)	16% (30)	36% (53)	87% (13)
Lung	48% (81)	49% (83)	24% (40)	15% (25)	27% (23)	90% (19)
Hematologic	50% (83)	52% (85)	28% (46)	17% (28)	27% (27)	75% (6)
Gynecologic	46% (64)	59% (83)	32% (45)	22% (31)	29% (26)	100% (4)
Colorectal	46% (50)	58% (63)	32% (35)	19% (21)	34% (25)	100% (7)
Head and Neck	58% (38)	47% (31)	24% (16)	13% (9)	24% (9)	100% (3)
Other	46% (127)	53% (145)	28% (78)	15% (42)	36% (59)	70% (14)
Cancer stage at diagnosis						
Stage 0‐II	51% (425) .177	54% (454) .430	30% (247) .250	19% (158) .070	37% (199) .001	81% (42) .0427
Stage III‐IV	49% (182)	53% (198)	27% (99)	16% (60)	24% (58)	92% (23)
Missing	44% (113)	50% (127)	25% (63)	13% (33)	30% (49)	87% (13)
Comorbidity score						
0‐1	48% (442) .485	50% (462) .017	26%(237) .051	16% (147) .303	29% (161) .004	83% (38) .684
≥2	51% (264)	58% (298)	32%(164)	19% (99)	38% (135)	86% (37)
Missing	45% (14)	61% (19)	26% (8)	16% (5)	48% (10)	100% (3)
Symptoms						
Fatigue	50% (613) .053	54% (660) .100	28% (342) .799	18% (215) .256	33% (263) .456	86% (67) .482
Pain	51% (441) .138	53% (463) .920	28% (242) .943	17% (146) .684	33% (186) .862	87% (48) .418
Breathing problems	52% (414) 0.036	53% (424) .807	28% (226) .799	18% (147) .179	36% (192) .010	88% (57) .228
Distress	51% (380) .115	56% (414) .044	28% (206) .879	19% (137) .175	34% (157) .516	84% (42) .820
Body mass index						
Underweight (<18.5)	47% (17) .004	53% (19) .003	17% (6) .018	11% (4) .002	00.0% (00)	100% (5) .645
Normal weight (18.5‐24.9)	46% (225)	47% (232)	24% (119)	13% (63)	0.00% (00)	80% (33)
Overweight (25.0‐29.9)	47% (247)	54% (285)	29% (152)	18% (93)	18% (94) <.001	88% (21)
Obese (≥30)	57% (231)	60% (243)	33% (132)	22% (91)	52% (212)	86% (19)
Godin Leisure‐Time Physical Activity (LTPA), Moderate or Vigorous Physical Activity						
<150 min per week	48% (574) .186	52% (621) .150	27% (325) .320	17% (200) .529	33% (255) .659	85% (70) .656
≥150 min per week	53% (146)	57% (158)	30% (84)	18% (51)	31% (51)	80% (8)
Current smoker						
Yes	45% (41) .336	50% (46) .050	26% (24) .666	13% (12) .270	17% (8) .023	88% (78)
No	50% (678)	54% (731)	28% (384)	18% (239)	33% (297)	—

Note

*P* values ≤ .028 are significant at a 10% False Discovery Rate level.

## 4 DISCUSSION

Among surveyed older cancer survivors, most reported discussing at least 1‐of‐3 HLS topics (exercise, healthy diet, or vegetable consumption) with a healthcare provider during or postcancer care, with less than one in five having discussed all‐3 topics. Daily vegetable consumption was the least commonly discussed topic. Weight loss among overweight or obese survivors was not frequently discussed either. Moreover, in this population, participants recalled discussions that occurred less frequently with nurses or oncologists than with other doctors. Furthermore, healthcare providers might be overlooking the need for HLS discussions with older, white, or longer‐term cancer survivors.

The frequency of HLS discussions among cancer survivors has been generally low. Among cancer survivors in the 2000 National Health Survey, only 26% reported discussing exercise, 30% diet, and 63% smoking cessation (among smokers).^10^ In a different study of California residents with cancer, 68% of survivors reported having discussed exercise, 61% diet, and 87% smoking cessation.^12^ Similarly, in a study limited to colorectal and lung cancer survivors from different parts of the country including Alabama, 59% of the respondents reported discussing exercise and 44% reported discussing diet.^11^ Among breast cancer survivors surveyed in Ohio, 53% discussed exercise and 37% nutrition and/or weight management.^14^ In a more recent study conducted in a large, university‐affiliated hospital in North Carolina, 35% of oncology clinicians reported communicating with their early stage breast, colon, and prostate cancer patients on exercise. Even though our sample was composed of older survivors, the findings from our study are in line with these studies, with about half of our study respondents discussing diet or exercise and 85% of respondents who were smokers discussing smoking cessation. Thus, the frequency of these discussions may have remained low despite strong recommendations from ASCO in recent years.^8^

The discussion of vegetable consumption was particularly infrequent in our study. Following healthy diets with an emphasis on plant‐based food consumption could protect from secondary cancers and decrease cancer related mortality.^6^, ^19^ It was recently found that consuming vegetables is especially important for diversifying gut microbiome, which in turn lowers chronic inflammation, pain, fatigue, improves immunity, decreases tumorigenesis, and potentiates immunotherapeutic effects in cancer treatment and prevention.^27^, ^28^

A concerning finding from our study is that only a third of overweight and obese cancer survivors reported discussing weight loss. Weight loss recommendations in older obese and overweight survivors could play a major role in decreasing cancer related mortality, secondary obesity‐related cancers, cancer recurrence, and comorbidities.^8^, ^19^, ^20^ More research should investigate the reasons why these discussions are so infrequent. Societal stigma and provider discomfort discussing the sensitive nature of this subject matter might be reasons for infrequent discussion that could potentially be addressed.^29^ Moreover, less than two in ten respondents reported having discussed all‐3 main HLS topics (exercise, healthy diet, and vegetable consumption) with their providers, and even fewer may have discussed them concurrently. This may be concerning, because delivering exercise and nutrition messages together may have stronger long‐term effects on weight management and other health outcomes compared to single message interventions.^30^ Future studies should investigate the prevalence of concurrent delivery of HLS discussions.

In this older cancer population, HLS discussions were initiated by nononcologists more frequently than by oncologists or nurses. A missed opportunity exists for oncologists in addressing survivors’ important needs related to healthy lifestyle modification during oncology clinic visits. Oncologists, as leaders in cancer survivors’ care, could play a critical role in encouraging and motivating survivors towards healthy behavior change. This finding also may be the result of a higher proportion of our sample being longer‐term survivors who more often recalled more recent conversations that occurred with their primary care physicians as compared to their cancer care team. Alternatively, oncologists and nurses may be uncomfortable holding these conversations due to lack of knowledge regarding HLS topics, time, motivation, discomfort, burnout or not believing in the importance of healthy behavior discussions during oncology clinic visits.^29^, ^31^ Lack of reimbursement mechanisms could be another barrier preventing oncologists from holding these conversations, as has been reported for primary care physicians.^32^ More in‐depth research studies exploring reasons why oncologists and nurses are not as frequently involved in HLS discussions could aid in understanding barriers, facilitators, and feasibility of following ACS HLS counseling guidelines in oncology clinics. These studies could guide development of tools, approaches, and models of care (eg technology, shared decision‐making approaches,^33^ integrative oncology care models^34^), assisting providers in incorporating HLS promotion in survivorship care^15^ in time‐constrained clinic environments.

Understanding reasons why older (≥75 years), white, or longer‐term survivors less often receive HLS counseling from their providers is important and should be further explored. Future multivariate analyses are warranted to identify what may confound the association of age, race/ethnicity, and time from diagnosis with HLS counseling, and better understand why these differences are observed. Moreover, it will be important to understand whether these differences are due to patients’ needs and preferences as opposed to provider‐related and/or healthcare system factors. The implications for how to intervene and optimize the frequency and quality of HLS discussions depend on this knowledge.

Although this is the first analysis, to our knowledge, that provides a comprehensive and multicenter evaluation of HLS counseling received by older cancer survivors in the southeastern US, there are limitations. Respondents received care at 12 cancer centers; thus, the findings may not be generalizable to the overall cancer population in this region or in the US population at large. Types of comorbidities, dietary habits, and amounts of alcohol consumed by cancer survivors in our study were not collected and therefore could not be analyzed. It also is possible that those not reporting HLS conversations with their providers were already following HLS. The study design (one‐time cross‐sectional survey) does not allow the evaluation of the longitudinal impact of HLS counseling services on cancer survivors’ health outcomes. This study relies on survivors’ self‐report, and there is also a possibility of recall bias. Further model‐based, adjusted analyses may be needed to account for confounding factors that might have affected the associations of study outcomes with patient characteristics, specifically age, race/ethnicity, time since cancer diagnosis, as well as with provider type.

## 5 CONCLUSION

The prevalence of HLS discussions among older survivors is suboptimal. Some healthcare providers might be missing the opportunity to have HLS discussions on important topics such as weight management and vegetable consumption, and to deliver this essential component of survivorship care.^15^ Additional research is needed to understand reasons for infrequent HLS discussions in oncology clinics, especially for some demographic groups, as well as patient needs and preferences for HLS counseling throughout cancer care.

## CONFLICT OF INTEREST

None.

## AUTHOR CONTRIBUTIONS

Conceptualization: KH, WD‐W, MP; Methodology: KH, WD‐W, MP, AA; Data Curation and Analysis: MP; Investigation: All; Writing – original: KH; Writing – review/edit: All; Supervision, project administration and funding acquisition: MP, WD‐W.
