# Supplementary material for: Healthy lifestyle discussions between healthcare providers and older cancer survivors: Data from 12 cancer centers in the Southeastern United States
Source: Cancer Med. 2019 Sep 30;8(16):7123–32. doi: 10.1002/cam4.2568 (PMC6853832; doi:10.1002/cam4.2568)
Supplement: Supplementary file 1 [file CAM4-8-7123-s001.docx]

Supplemental Table 1. Healthy lifestyle (HLS) counseling questions used in a survey of cancer survivors at 12 cancer centers in Southeastern U.S. from November 2013 to June 2015.

At any time during your cancer care and after, has your provider, or anybody else on your care team, advised you to…

- Exercise (other than post-surgery rehabilitation exercises)?
  - If yes, who advised you to exercise? Was it your: (check all that apply)

a) Oncologist; b) Another doctor c) Nurse

- Follow a healthy diet (low in animal fat, sugar, processed foods)?
  - If yes, who advised you to follow a healthy diet? Was it your: (check all that apply)

a) Oncologist; b) Another doctor c) Nurse

- Eat 5 or more servings of vegetables per day?
  - If yes, who advised you to eat vegetables? Was it your: (check all that apply)

a) Oncologist; b) Another doctor c) Nurse

- Lose weight (if overweight or obese; BMI ≥25)?
  - If yes, who advised you to lose weight? Was it your: (check all that apply)

a) Oncologist; b) Another doctor c) Nurse

- Quit smoking (if currently smoking)?
  - If yes, who advised you to quit smoking? Was it your: (check all that apply)

a) Oncologist; b) Another doctor; c) Nurse
